# Veterinarians and Humane Endings: When Is It the Right Time to Euthanize a Companion Animal?

**DOI:** 10.3389/fvets.2017.00045

**Published:** 2017-04-19

**Authors:** Oliver Knesl, Benjamin L. Hart, Aubrey H. Fine, Leslie Cooper, Emily Patterson-Kane, Kendall Elizabeth Houlihan, Raymond Anthony

**Affiliations:** ^1^Zoetis Inc., Parsippany, NJ, USA; ^2^Department of Anatomy, Physiology and Cell Biology, School of Veterinary Medicine, University of California Davis, Davis, CA, USA; ^3^College of Education and Integrative Studies, California State Polytechnic University, Pomona, CA, USA; ^4^Davis, CA, USA; ^5^Animal Welfare Division, American Veterinary Medical Association, Schaumburg, IL, USA; ^6^Department of Philosophy, University of Alaska Anchorage, Anchorage, AK, USA

**Keywords:** companion animal, euthanasia, human–animal bond, humane endings, veterinary medicine, wellness

## Abstract

Current advances in technologies and treatments provide pet owners and veterinarians with more options for prolonging the life of beloved pets, but can simultaneously lead to ethical dilemmas relating to what is best for both animal and owner. Key tools for improving end-of-life outcomes include (1) sufficient training to understand the valid ethical approaches to determining when euthanasia is appropriate, (2) regular training in client communication skills, and (3) a standard end-of-life protocol that includes the use of quality of life assessment tools, euthanasia consent forms, and pet owner resources for coping with the loss of a pet. Using these tools will improve outcomes for animals and their owners and reduce the heavy burden of stress and burnout currently being experienced by the veterinary profession.

## Introduction

The changing nature of the bond between humans and animals has made pets an increasingly integral part of many people’s lives. This in turn has changed the landscape for the veterinary profession such that the human–animal bond must be integrated into daily veterinary care (e.g., “bond-centered” practice) (1, 2). At the same time, veterinary medicine has developed a wide range of technologies and treatment options, with the result that veterinarians and pet owners are increasingly confronted with ethical dilemmas concerning whether a procedure that is medically available is truly appropriate for the animal or the owner. Recognizing that there is a diversity of cultures and beliefs influencing the veterinarian and the owner, the veterinarian’s core duty remains to navigate these in order to serve the best interests of the animal and owner.

The following commentary looks specifically at the decision-making process in relation to the euthanasia of a companion animal and asserts that this needs to be supported by specific tools that assist the veterinarian in serving the interests of the animal and the wellness of the client and veterinarian.

## Euthanasia Decisions are Stressful for Veterinarians

It has been clearly established that euthanizing animals is broadly stressful for veterinarians (3). This includes the euthanasia of companion animals that are unwanted in shelters or at owner request a.k.a. “convenience” euthanasia (4). This article, however, focuses on serving clients bonded to animals with terminal conditions that cause suffering, where euthanasia should be considered. Discussions of this type are a regular feature of many veterinarians’ days and the stress they cause may be exacerbated by veterinarians not easily accepting death as an outcome. That is: “*Veterinarians are trained to heal, but they are routinely confronted with ending life rather than saving it*”.^1^ As “gatekeepers” for the animal’s end of life, veterinarians find themselves acting on behalf of the animal’s silent voice (5), and needing to somehow also accommodate the needs and desires of their human clients, and a plethora of idiosyncratic circumstances that limit their therapeutic and emotional resources in that moment.

In one study of 21 veterinary students, several pointed out numerous distressful situations that they had already experienced (6). Even at this stage in their training, the highest level of stress related to end-of-life decisions. One student described that his major sense of stress occurred when he believed an animal’s suffering was prolonged because the owner did not want to accept that its condition was not likely to improve and that death was inevitable. The student went on to state that: “*Sometimes we take cases too far and subject dogs to radiation or chemotherapy to satisfy the client* …. *A lot of times the animal is in intensive care and it’s just for the people*.” This scenario was also reported as a common and stressful dilemma for practicing veterinarians (7).

Although euthanasia serves as a method to end suffering in animals, the decision, inevitably, is difficult, and highly bonded owners require more support from their veterinarian at this time (8). Most committed pet owners have probably experienced, or know someone who has experienced, a situation where they were left with a feeling that euthanasia was provided for an animal too soon or delayed too long. Both owner and veterinarian have some ability to refuse euthanasia which may cause it to be withheld when it would serve its role in ending unnecessary suffering, and both client and veterinarian may feel guilt for being responsible for an animal’s death (8–10).

The ethical and emotional strains associated with euthanasia decisions are often not openly acknowledged or a subject of structured assessment and improvement efforts, despite their importance to the veterinary profession and pet owners (9). Few veterinarians could name specific tools they use to define and address these problems, and their prior education may not have included any instruction on these tools (7, 9, 11). The first additions to this tool kit, if not already acquired, should be an *ethical decision-making framework*, a good *communication system*, and *quality of life assessment and consent tools*.

### Ethical Decision-Making Frameworks

Engaging in a quality process, e.g., training in ethical deliberation, can have the effect of minimizing distress experienced by veterinarians as a result of routinely performing euthanasia, one of the major reasons for burnout and moral distress (12). By adopting conscientious ethical decision frameworks that focus on the animal and its interest, less emphasis is placed on the question: “When is the right time?” with which veterinarians are routinely confronted and which can often exacerbate moral distress. Ethical decision-making frameworks that encourage deliberation, dialogue, and agreement between owners and veterinarians on important characteristics of quality of life for the animal patient in question engenders trust between veterinarians and their clients. By promoting trust and effective communication as essential skills to ensure healthy veterinarian–client partnerships, the veterinarian can devote their attention to making sure that the interests of a dying animal are prioritized.

By emphasizing the quality of the deliberative process (including acknowledging the role of animal-based measures in guiding the decision to euthanize and when), and not merely focusing on the final outcome, veterinarians can have confidence that they are living up to their commitment to provide quality care to their animal patients at the margins of life and that they have provided conscientious counseling to their clients, integrating their clients’ needs and interests into the deliberative process (5, 13).

In Sandoe et al. (14), veterinarians can find a robust analysis of the ethical principles that typically factor into the deliberations that deal with companion animal palliative care and euthanasia. Here, the authors conclude by stressing that while the interests of the pet owner, veterinarian, and animal may not always be fully compatible, it is the quality rather than the quantity of the animals’ life that is of greatest ethical significance. For some pet owners and veterinarians, the decision to euthanize an animal can be made rationally on the basis of financial, convenience, or compassionate considerations. For others, attachment to an animal can significantly complicate the decision as can the inherent inability of animals to communicate their preference. The later situation resembles that seen with very young, mentally impaired, or comatose human patients, where the principle of “best interests” can be applied to the decision-making process. This implies that the decision should always consider what is best for the animal, irrespective of the owner’s wishes. An opposing viewpoint is one that focuses on the best interest of the owner (a contractarian perspective) and can lead to situations where a suffering animal is kept alive to minimize the emotional distress of the owner. Veterinarians frequently have to search for a middle ground between the extremes of keeping an animal alive because modern veterinary medicine provides the means to do so (overtreatment) or situations where the owner insists that everything humanly possible must be done (contractarian) and considering what is in the best interests of the animal (14).

#### Rights and Care Considerations

Although there are numerous ethical models that represent a spectrum of ethical problem solving alternatives (e.g., utilitarian, virtue, and fairness approaches), the authors will focus on a couple of principles from two alternatives. Veterinarians may wish to begin their ethical deliberations by considering two basic principles. Firstly, according to the ethics-of-rights approach (15), an ethical action is an alternative that protects and respects the rights of all parties involved (5). This principle suggests that veterinarians have a primary obligation of respecting the ethical rights of individuals, both the animal and the client, and where necessary striving to resolve conflicts between the two (i.e., to be “fair”). This principle assists in considering what constitutes a valid right and how the rights of stakeholders should be balanced.

The ethics-of-care approach (16) places greater emphasis on the relationships and bonds that individuals have with each other. According to this approach, decision making should be guided by a motivation to care for dependent and vulnerable beings. This includes the veterinarian’s primary duty to the patient, and how the patient’s health impairments impact quality of life (5, 13). Tools based on this principle can assist veterinarians in finding ways to help clients be guided by their own duty of care as an animal owner and caregiver.

Veterinarians tend to innately understand both principles. For example, one study found veterinary students approached hypothetical ethical dilemmas trying to achieve a fair outcome for all which and a care-centered approach, being empathetic to the companion animal and human caregiver (17).

#### Deliberate Frameworks

In terms of a deliberate framework to guide veterinary medical interventions, two valuable models are provided by Morgan (18) and van Herten (19). These frameworks focus on case-by-case care; they begin with the interests of the animal patient as their starting point and are set within the framework of deliberation. This deliberation is a structured process that is investigative by nature and which invites partnership between the veterinarian and the owner or client to reach a shared treatment outcome for the animal patient. The frameworks give veterinarians the opportunity to discover their own values with respect to veterinary medicine and quality of life issues (value articulation) and to employ this knowledge in determining the best course of treatment for their animals, in conversation (and hence with some measure of transparency) with the client (moral deliberation) (20). The process of identifying central issues (e.g., medical indications) and the underlying ethical values or principles at work [e.g., respect for client’s autonomy, beneficence, non-maleficence, and justice (11, 17, 21)] is coupled with careful dialog about reasonable treatment alternatives (informed by ethical detective work and backed by empirical evidence), limits of technology, the capacity of both veterinary medicine and client husbandry to maintain the quality of life for the animal patient, and what responsible companion animal ownership entails at the end of life for this animal. These deliberative frameworks help the veterinarian find common ground with the client, and acknowledge the roles of the veterinarian as information and service provider and as animal and client advocate (22). As van Herten notes “*Whether killing is in the interest of the animal itself and its future well-being and whether there are human interests that outweigh the interest of the concerned animal to stay alive* … *the veterinarian and owner must find morally acceptable justification for euthanasia*.”

These frameworks have the additional effect of helping clients prepare for their companion’s passing while honoring the human–animal bond. Empathy central in the process of deliberation. However, if excessive, it can also increase the stress experienced by the veterinarian without improving the outcome (23).

By going through the assessment and deliberative framework highlighted here, veterinarians have the opportunity to communicate with their clients in a way that helps expose and gently resolve conflicts whenever possible, for example, where the medical evidence shows that an animal’s condition is terminal, but the client is still seeking therapeutic treatment; or when clients need to keep the animal in their life but the animal’s quality of life has reached the point where it does not have a life worth living. It may also occur that the animal’s life could potentially be extended from a medical point of view, but this option is not truly available because the owner has an unresolvable obstacle to supporting that option (24).

When veterinarians are able to explicate their own ethical roadmap confidently, they will also be able to reliably guide a client toward a sound decision and leave them feeling they made that decision in a morally sound way. The two frameworks highlighted here reflect the deliberative and assessment guidelines regarding the morality of euthanasia that can be found in the introductory section of the American Veterinary Medical Association’s (AVMA) *Guidelines for the Euthanasia of Animals* which also includes some ethic decision aids for euthanasia (25). These frameworks build public confidence in the profession that “the delicate task of weighing the request to perform euthanasia and to give owner advice in the end of life decisions” (19) are made judiciously and that the ethical process is shaped around the goal of providing a good death for companion animals. Furthermore, one of the virtues of the process’ quality is that it is done in a transparent manner, highlighting how veterinarians are fulfilling their obligations to their clients, animal patients, and the public (25).

One example of a specific tool consistent with these principles is available from the Markkula Center for Applied Ethics, Santa Clara University. The tool provides a simple framework for ethical decision making that can be accessed *via* their website or downloaded as an app.^2^ The app helps users ask questions about ethical choices and can serve as a good starting point for exploring ethical decision-making.

Once clients develop a high level of understanding of the ethically acceptable options, veterinarians may be prompted to expand which options they can make available through continuing education, the use of consultants, or referrals. Veterinary options have increased not only in the area of treatments and techniques but also the manner in which these can be made available to clients (e.g., client training, mobile practice, and veterinary hospice). These caregivers should be folded into the communication system discussed below. Sandoe et al. (14) highlight “overtreatment” as an ethical concern that also needs to be considered given the increasing availability of advanced treatment options and pet insurance. The potential for overtreatment is a particular concern in certain veterinary specialties (geriatric medicine, oncology, emergency and critical care) where seriously ill animals may be “saved” but where the negative experiences of continuing to live outweigh the positive.

Explicit ethical discussion may also expose less-than-optimal client–veterinarian pairings; for example, clients from religions that do not permit euthanasia may not fit well with a veterinarian unable to accept and work with this perspective. As with physicians or other professional caregivers, while every effort should be made to meet client needs, clients should also be supported in selecting a practitioner that best meets all of their needs rather than choosing them purely on an arbitrary basis of proximity, habit, fear of causing offense, or the desire to retain a customer client.

### A Communication System

Given the intricacies of caring for pets and their families during humane endings, robust relationships between the veterinary team, client, and patient can reinforce and maintain the veterinarian’s role as a trusted guide even when unexpected adverse events occur. Conversely, when a companion animal end-of-life experience is poorly handled and this trust is not present, ongoing personal guilt and recrimination can reverberate for the pet owner and grief may become more debilitating (8). This may lead to a client going to another veterinarian, avoiding further veterinary care, or ceasing to be a pet owner. These relationships are built over time through empathic communication.

In many busy veterinary practices, clients only consult a veterinarian when their pets need medical attention and frequently they see whichever veterinarian happens to be available at the time. This approach of treating veterinarians as interchangeable may be implicitly supported when the practice does not clearly record and seek to reconnect clients with their regular veterinarian, and thus undermines the veterinarian–client relationship (10, 26).

However, regular contact is not sufficient to ensure that a good relationship will develop. Borden et al. (27) analyzed euthanasia discussions in companion animal practice and found that some veterinarians were not fully exploring client feelings, ideas, and expectations. Additionally, they also found that many veterinarians did not always solicit client involvement in defining the problem and in developing goals for treatment and management of the pet, particularly with less assertive clients. Explicit training in communication will assist veterinarians in providing the support required to make ethical decisions and help ameliorate feelings of guilt or grief.

Communication skills are eminently teachable (28, 29) and specific communication skills can be learned and implemented in a veterinary practice with great success (30, 31). Appropriate training programs and workshops, such as the frank™ Communication Series (see text footnote 1) can improve the communication skills of all veterinary practice staff. Training in more than one method will assist veterinarians in developing formalized systems most suitable for their practice and provide options for adapting their approach to the needs of the client. Effective veterinary practitioners continue perfecting their communication skills in non-verbal communication, reflective listening, open-ended inquiry, and empathic statements throughout their careers, and mentoring others in these skills (28).

Development and discussion of communication tools and skill sets by the veterinary team as a whole can also help in acknowledging the stress associated with euthanasia decisions for both the owner and staff involved in the patient’s care. One appropriate approach is considering client relationships and emotions as topics for continuing education and team communication during regular staff meetings. This can improve staff interactions with clients and encourage the development of healthy coping mechanisms as part of an overall staff health and wellness plan.

Deciding how to manage a pet near the end of its life can be a very difficult process, and a pre-established habit of honest, respectful, and caring communication reduces stress and the chances of misunderstandings. Engaging established clients in conversations about quality of life and gradually introducing the idea of end-of-life goals for their pets prior to the time when those decisions are needed helps them establish appropriate goals and expectations prior to a time when the pet is already in crisis and the owner’s grief process has been triggered by the realization that their pet has a terminal condition.

Without effective client communication, clients may not be given the time or discussion they need to transition away from therapeutic treatment and toward palliative care, or they may not even realize this has occurred. Clients may inadvertently feel pressured into end-of-life decisions or miss the chance to make decisions about the euthanasia that may differ from the practices more commonly selected, i.e., “default” opinions. For example, it used to be assumed clients should not observe the euthanasia, whereas when clients make this decision for themselves, the majority opted to be present and this was often helpful to them in the grief process (32). Practices that are the norm today may be similarly misguided, and we rely on clients to let us know when they are not comfortable with what is happening.

### Quality of Life Assessment Tools

One specific example of a tool to use during discussions with clients is a quality-of-life assessment. These assessments are like other health and welfare questionnaires or checklist tools for healthy animals (33) but are focused particularly on the needs of animals during the end-of-life period. They help clients appreciate the severity of the health problems their animals are experiencing and how this is affecting their ability to enjoy life. Ideally, the owner and veterinary team can use these to track the progression of the animal’s condition and agree on a point where euthanasia should occur. These tools direct clients to focus on their animal’s quality of life as being the key determinant for when euthanasia becomes ethically appropriate. Veterinarians and their staff play a pivotal role in communicating the medical realities of a pet’s disease to pet owners, while simultaneously respecting the strong emotional bond between a client and their pet (34).

Quality-of-life tools vary in the circumstances they address, and veterinarians should be familiar with or seek out examples suitable for each patient and client. There are general checklists that might help illuminate whether any animal has “a life worth living” (35); there are species-specific tools that address the effect of a particular disorder or symptoms (36–38). Villalobos and Kaplan (39) have proposed specific parameters for caregivers of cancer patients. These include the degree of pain the pet is experiencing, changes in mobility, presence of appetite, hydration, and an estimation of the proportion of “good” versus “bad” days. This can help a client to more objectively monitor a pet’s quality of life. If individual scores reach only 30–50% of normal for a pet, this could be set in advance as the point at which a decision to euthanize is taken. Bijsmans et al. (40) have validated a psychometric tool for assessment of owner-perceived quality of life in cats and used it to compare quality of life between healthy cats and those with chronic kidney disease.

Veterinary hospice and palliative care are currently hindered by an inadequate amount of scholarly research to guide clinicians. The increasing use of palliative care and hospice in veterinary medicine necessitates further development of quality-of-life assessment tools for decision making when caring for terminally ill pets (41). The 2016 AAHA/IAAHPC End-of-Life Care Guidelines for Dogs and Cats review the latest information to help staff address central issues and perform essential tasks to improve the quality of life of a pet that has entered the final life stage.^3^ The guidelines include resources to assist in client communication and patient care.

For vets beginning to use these tools for the first time, a practical example of a simple pet owner quality of life assessment tool is provided by The Honoring the Bond service at the Ohio State College of Veterinary Medicine.^4^ This service also provides a Pet Loss Library containing various resources for pet owners on how to make end-of-life decisions and cope with the loss of a pet. The quality-of-life assessment tool is a printable brochure titled “*How Do I Know When it’s Time? Assessing Quality of Life for Your Companion Animal and Making End-of-Life Decisions*”^5^ that provides pet owners with a suite of parameters to help assess their pet’s quality of life in conjunction with a veterinarian. Another important resource offered through the Honoring the Bond service is access to a veterinary social worker who can act as a liaison between the animal owner and veterinary medical team.

When a decision to perform euthanasia is reached, it should be expressed clearly both verbally and in writing, such as the Model Euthanasia Authorization^6^ developed by the AVMA. The use of euphemism and verbal-only communication is subject to misinterpretation. The client should then be educated about the process and options and given as much control over the process as is practicable and consistent with ethical practice.

## The Problem of Veterinary Burnout

As we have discussed, everyone associated with an animal’s death can be strongly impacted, including veterinarians (42). The impact for veterinarians dealing with sick and dying patients on a daily basis can eventually take its toll, resulting in tremendous emotional stress and the well-recognized “burnout” syndrome. The topic of burnout is a great concern in human medicine, and there is evidence that veterinarians in practice are affected the same way (43). Aside from the adverse impact on the mental health of practitioners, burnout could be considered the “elephant in the room” with regard to effectiveness in communication with clients (1).

A full 50% of medical clinicians—and most likely veterinary clinicians as well—suffer burnout, characterized by emotional exhaustion, cynicism, and negative self-evaluation^7^ (44). Hartnack et al. (11) found younger female veterinarians and those working in small or mixed animal practice to be at a higher risk of work-related stress and suicidal thoughts. Burnout may lead to a loss of empathy with clients and attentiveness to their needs, resulting in clients being less compliant with treatment instructions (45, 46). Additionally, veterinarians experiencing burnout may appear withdrawn or uncaring to their clients.

Strategies for preventing or dealing with burnout range from insisting on meaningful breaks from practice, such as vacations with no email or phone access to the hospital, a regular exercise program, colleague support group meetings, and opportunities for mindfulness and meditation (47, 48). Aside from the mental health benefits to the clinician, one study in human medicine reported that clinicians rated as more mindful and engaged in more patient-centered communication had a higher percentage of satisfied patients (49). One expects that the findings on human patient communication apply equally to client communication in the veterinary realm—and this includes the reality of clinician burnout.

As well as seeking to cope with stress, efforts should also be made to reduce stress to its lowest necessary levels. There is evidence that veterinarians cope better with stress resulting from adverse surgical events when they can learn something from the experience that can benefit their future patients (50). This same approach of open discussion and constructive learning should be taken to both euthanasia decision making and the euthanasia process, from the decision point to the disposition of the remains. It has been shown that veterinarians benefit from having colleagues at work who discuss cases and provide mutual support during and after euthanasia (11). Veterinarians who lack this support within their workplace can look for safe and supportive veterinary groups outside the workplace or online (e.g., “Not One More Vet” on Facebook).

If veterinarians ultimately find their employment does not or will not support them with an ethical decision-making framework or workplace wellness efforts, they should consider seeking out a different employment opportunity that will make it easier for them to find meaning and satisfaction in their work—as this is the greatest single factor for reducing workplace stress. Employers finding that they struggle to retain veterinarians and/or clients should include ethical and wellness considerations in their analysis of the problem.

The high rate of suicide among veterinarians (as well as human health professionals) is a matter of extreme concern (51), and a wide range of professional groups is attempting to address it. Pioneering work by Bartram and Baldwin (52, 53) led to the creation of the supportive Vetlife website^8^ in the United Kingdom. In the United States, assisted by the AVMA, concerned veterinary students have recorded brief videos expressing support to distressed fellow students, entitled “It’s OK” (54). The AVMA Future Leaders Program has developed tools that have been integrated in the AVMA’s Wellness and Peer Assistance resources.^9^ Volunteer positions are available for veterinarians motivated to help tackle this wellness crisis in the profession at a leadership level.

## Conclusions

Promoting healthy decision making during the euthanasia process of a companion animal is important for veterinarians’ wellness, for ensuring a humane and respectful outcome for animal patients, for strengthening the human–animal bond, and for maintaining the reputation of the profession. Veterinarians need to develop and maintain empathic, professional relationships with their clients if they are to serve as a partner in dialog when euthanasia is an alternative to continued suffering. Understanding the nature of the bond between the pet and caregiver plays the most important role in determining the outcome for each animal, owner, and veterinarian. Ideally, the occasion includes a shared and compassionate appreciation of the situation, the options, and the likely outcomes of the choices that are to be made. Veterinarians who are not comfortable with addressing the full range of a client’s needs should explore the options for training and collaboration with professionals trained in grief support.

The continued strengthening of the human–animal bond has greatly complicated the ethical conundrum surrounding euthanasia. Euthanasia is an emotional, psychological, and economic issue that every veterinarian must wrestle with. Many in the profession experience extensive stress as they grapple with the conundrum of ending an animal’s life too soon, or waiting too long, in addition to managing the client’s expectations and accommodating their emotional needs. A number of approaches are available to help veterinarians develop strategies for putting end-of-life decisions and experiences into perspective and to help prevent or deal with “burnout” syndrome. Having a working knowledge of ethical values, principles and decision-making frameworks can help veterinarians make decisions with confidence and in turn help their clients problem-solve and confront ethical dilemmas together.

For veterinarians wrestling with the conundrum of euthanasia, this article has outlined a number of ethical decision-making tools and deliberative frameworks that can to help them better manage end-of-life discussions with their clients to produce optimal outcomes. Actively encouraging clients to carry out regular, routine preventive care visits with the same veterinarian will foster the development of meaningful, long-term veterinarian–client–patient relationships. These relationships can be further enhanced when the veterinarian and all of the veterinary hospital staff undergo regular continuing education on client communication focusing on non-verbal communication, reflective listening, open-ended inquiry, and empathic statements. The ethical decision-making frameworks highlighted here facilitate conscientious, humane, end-of-life decisions between veterinarians and their clients in order to promote the best quality of life for animal patients. Finally, it is important for veterinarians to have a standard end-of-life protocol that includes the use of quality-of-life assessment tools, euthanasia consent forms, and pet owner resources for coping with the loss of a pet and that is reviewed at least annually with all of the practice staff.

## Author Contributions

All the authors contributed significantly to the concept and text.

## Conflict of Interest Statement

The authors declare that the research was conducted in the absence of any commercial or financial relationships that could be construed as a potential conflict of interest.

## Footnotes

^1^Verdon DR. *Euthanasia’s Moral Stress: A High Psychological Price*. DVM 360. (2003). Available from: http://veterinarynews.dvm360.com/euthanasias-moral-stress-high-psychological-price.

^2^http://www.scu.edu/ethics/practicing/decision/framework.html.

^3^https://www.aaha.org/graphics/original/professional/resources/guidelines/2016_aaha_iaahpc_eolc_guidelines.pdf.

^4^http://vet.osu.edu/vmc/companion/our-services/honoring-bond-support-animal-owners.

^5^http://vet.osu.edu/vmc/sites/default/files/import/assets/pdf/hospital/companionAnimals/HonoringtheBond/HowDoIKnowWhen.pdf.

^6^https://www.avma.org/KB/Policies/Documents/model-euth-auth-form.pdf.

^7^http://www.scu.edu/ethics/practicing/decision/framework.html.

^8^www.vetlife.org.uk.

^9^https://www.avma.org/professionaldevelopment/personal/peerandwellness/pages/default.aspx.
